# A Position Sensing Glove to Aid Ankle-Foot Orthosis Diagnosis and Treatment

**DOI:** 10.3390/s21196631

**Published:** 2021-10-06

**Authors:** Eduard Cazacu, Coen van der Grinten, Jeroen Bax, Guus Baeten, Fred Holtkamp, Chris Lee

**Affiliations:** 1 Fontys Institute of Engineering, Fontys University of Applied Sciences, 5612 AR Eindhoven, The Netherlands; ec@eduardcazacu.com (E.C.); coen@vd-grinten.nl (C.v.d.G.); jeroen152000@hotmail.nl (J.B.); guus.baeten@gmail.com (G.B.); 2 Fontys School for Allied Health Professions, Fontys University of Applied Sciences, 5612 AR Eindhoven, The Netherlands; f.holtkamp@fontys.nl

**Keywords:** ankle-foot orthosis, position sensors, smart glove, 3D modeling

## Abstract

A position sensing glove called SmartScan, which creates a 3D virtual model of a real object, is presented. The data from the glove is processed by a volume minimization algorithm to validate the position sensor data. This allows only data from the object’s surface to be retained. The data validation algorithm allows the user to progressively improve an image by repeatedly moving their hand over the object. In addition, the user can choose their own balance between feature resolution and invalid data rejection. The SmartScan glove is tested on a foot model and is shown to be robust against motion artifacts, having a mean accuracy of 2.9 mm (compared to a 3D model generated from optical imaging) without calibration.

## 1. Introduction

It has been estimated that about 57% of the Dutch population will suffer from foot problems at some point in their lives [1]. This figure is broadly representative of western European nations [2]. Magnusson et al. report that about 2.2% of the population of India have some kind of physical disability [3], including ankle-foot orthoses. Despite the relative prevalence of ankle-foot orthosis, successful treatment is inefficient, with about 20% of corrective measures failing to reduce suffering [4].

The high failure rate of ankle-foot orthosis is not due to the lack of skill or training, but rather, the lack of quantitative data [5]. The current standard involves the clinician taking a (plaster) cast of the foot while holding it in the corrected position. The clinician has no quantitative data about the correction and must rely on experience to obtain an accurate cast of the corrected foot. In addition to the lack of data, the process is wasteful—each foot correction requires a plaster cast—and slow, as the cast must be shipped to another location for processing.

To turn ankle-foot orthosis into a data-driven process, we propose the creation of virtual foot models that can be manipulated by the clinician in order to better control and test different possible corrections. To achieve this, a near-real-time virtual model of the foot and the proposed corrections needs to created. After examining the digital model, the model could be locally fabricated (using 3D printing) for testing, before a more durable corrective insert is manufactured. The proposed workflow would shorten the feedback path and allow the clinician to comparatively quickly find the most appropriate corrective measure.

A virtual model of a foot can be easily created using laser scanning technology [6]. Laser scanning is highly accurate and rapid. The rapidity of laser scanning eliminates motion artifacts. Unfortunately, if the clinician manipulates the foot (an integral part of diagnosis) during imaging, the manipulation is obscured by the clinician’s hands. Thus, it becomes difficult to extract a virtual model of the manipulated foot. Roberts et al. reported an increased delay between the initial scan measurements and successful treatment [6].

It is now possible to use a phone camera or table camera to create a 3D model of a foot. However, Farhan et al. found that the quality of evidence that 3D scanning techniques, including laser scanning, improve healthcare outcomes is weak [7]. Although the reasons for the lack of improvement do not appear to be well investigated, some possible explanations include distortions due to motion artifacts and that the images include the hands of the clinician, obscuring the manipulated foot, and preventing the creation of an accurate determination of the required mould.

Here, we propose SmartScan, a glove that allows the clinician to create a 3D virtual model of the foot by feeling and manipulating the foot. The advantage of the smart glove solution is that the hand is the primary diagnostic tool of the clinician. By using sensors in the glove to create the model, it is possible to integrate virtual model creation into the standard workflow of the clinician. Furthermore, as the clinician manipulates the foot, a smart glove may record these manipulations, allowing for further analysis and to use this record to train others. In addition, SmartScan is applicable to all forms of ankle foot orthosis, independent of their cause, because SmartScan is a measurement technique rather than an independent diagnostic tool. However, no such glove exists.

The use of smart gloves in virtual reality is common. Gloves with pressure sensors and accelerometers are commonly used to translate real-world object manipulation into virtual reality [8]. However, the object to be manipulated is often pre-programmed into the environment.

Similarly, smart gloves can be used to virtually sculpt objects [9,10,11]. In these cases, a virtual object is manipulated using a smart glove that has position sensors and/or accelerometers. Unlike the virtual reality model above, no real object exists and motion is translated purely into the manipulation of a virtual object.

To our knowledge, there is no system that generates a virtual object from the motion of a smart glove around a real object. For the diagnosis and treatment of ankle-foot orthosis, such a glove may be a significant step in improving treatment success rates. A smart glove can be used to recreate a model of the foot, including the way that the clinician moulds the shape of the foot. The clinician can then use virtual reality or computer aided design (CAD) software to manipulate the foot model and judge which corrections are most appropriate. From there, a CAD model of the required mould can be created and manufactured using 3D printing, speeding up treatment and reducing waste.

In this paper, we present a proof-of-concept of SmartScan—a glove fitted with position sensors. We present the data processing algorithm that is used to validate the position data and generate an accurate 3D model of an object that is manipulated by the user. We find that the accuracy of the SmartScan point cloud is comparable to that of an optical scanner.

## 2. Materials and Methods

### 2.1. Hardware

**Figure 1:**
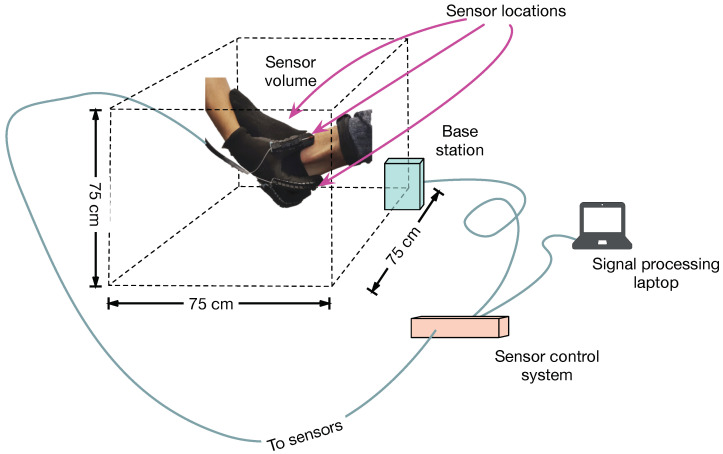
Ilustration of the physical setup. All measurements are referenced to the center point of the base station (light blue). The base station and sensors are connected to a control system (tan box) via cables. The sensors are mounted in a glove as shown, and at the location indicated on the foot (sensor not shown). The sensor volume is given by the dashed lines. The sensor data is processed by a computer for real time display.

Position sensors (NDI 3D Guidance model 180) with six degrees of freedom are mounted on a glove. The sensors use an electromagnetic field reference system (by measuring phase, amplitude and polarization relative to a reference, the three dimensional location and orientation of the sensor can be calculated relative to the reference location). The commercial sensor system is capable of measuring locations within a volume of 75 cm per side with a position accuracy of 1.4 mm (RMS) and an angular accuracy of 0.5${}^{\circ }$ (RMS) and at a sample rate of up to 2 kHz. Although the system is capable of sampling up to 25 position sensors, for the prototype, three sensors are mounted on the glove (one on the thumb, one on the forefinger and one on the middle finger). A fourth sensor is mounted on the object of interest to counteract motion artifacts (see Figure 1).

In using SmartScan, the user is required to provide the system with at least one reference point. Thus, before “scanning” the object, the user first uses the glove to define a series of reference points (see Section 2.2) and then begins the scan.

### 2.2. Software

**Figure 2:**
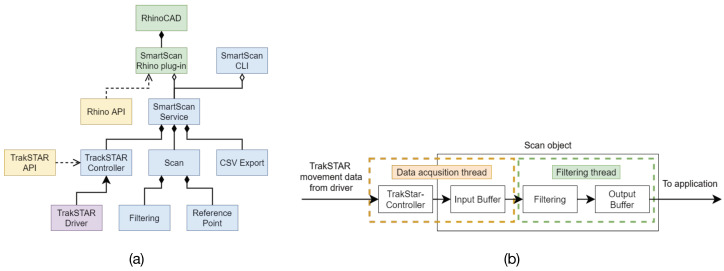
Software architecture (**a**). Green: RhinoCAD, SmartScan Rhino plug-in; blue: SmartScanService, TrakSTAR controller, scan, CSVExport, filtering, ReferencePoint; yellow: RhinoAPI, TrakSTAR API; purple: TrakSTAR driver. Data processing pipeline (**b**). Yellow: acquisition thread; green: filtering thread.

The sensor data is transmitted to a laptop where a service (see Figure 2a and Table 1) receives and processes the data. The data processing pipeline, shown in Figure 2b, consists of two threads: one dedicated to receiving and storing the data in memory, while the other is devoted to filtering. The filtered data is then either transferred to the CAD software or is stored to disk in a comma-separated value file. The service is implemented so that it can run as a standalone application or as a plugin in the CAD software system.

The position data from the sensor are translated and rotated to the reference frame of the reference sensor. In this way, if the object of interest moves during the scan, the image is not distorted.

When using the software, the user is asked to define reference points which represent the average location of all the sensors (excluding the reference sensor) for a fixed period of time. The user may define as many reference points as required. The role and requirements of the reference points are explained more fully in Section 2.3.

### 2.3. Filter Algorithm

Although the position sensors are highly accurate, SmartScan does not have any intrinsic knowledge of its environment. The data from the sensors report the position irrespective of whether the user’s fingers are touching the object of interest or not. Therefore, it is necessary to select the data that correspond to when the user is touching the object of interest. Furthermore, because it is desirable that the clinician not be required to change their behavior, the selection algorithm should operate with minimal user interaction.

Filtering is accomplished in two steps. The first step is a volume minimization filter, which, based on the location of a reference point, selects only those points that lie on a surface that encloses the reference location in the smallest volume. The second step searches for, and removes, outliers that the first filtering step misses.

**Figure 3:**
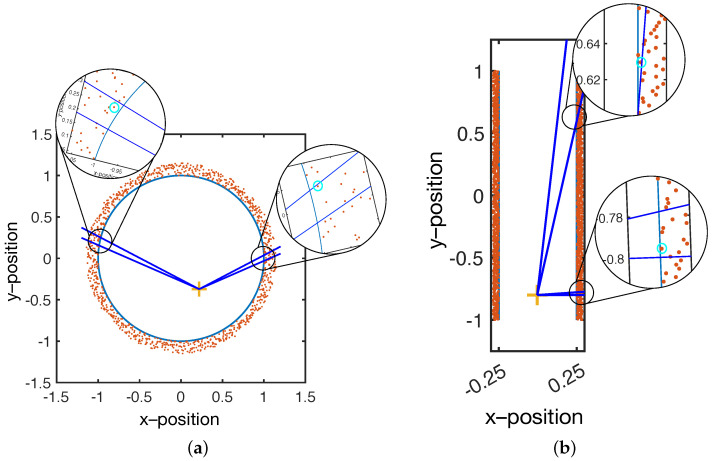
Single reference point in a spherical volume (**a**). Within the angular resolution (dark blue lines), only the point closest to the reference point (highlighted by the cyan circles in the insets) are passed on for visualization. With a near–spherical object, the areal density of points after filtering is nearly constant. Single reference point in a cylindrical volume (**b**). As with the case for the spherical volume, only the closest point to the reference point is selected. However, for some locations, the areal density of points is significantly reduced. In addition, due to the highly oblique angle, the selected point is not necessarily close to the surface (cyan circle top inset).

Volume minimization is illustrated in Figure 3. In short, SmartScan is used to scan a solid object; therefore, it is expected that there is a volume in which there are no position data points. Through a sensible choice of glove position, the user can ensure that the reference data points are located within the object’s volume.

As shown in Figure 3, the reference point acts as an origin, and the volume is divided into solid angular sections with a user-specified angular size. Within each section, only the points closest to the origin are selected, and the rest are discarded. Figure 3a shows that for a spherical (or near spherical object), a single well-chosen reference point is sufficient to select the sensor positions that correspond to the surface. Furthermore, the spacing between the selected positions will remain relatively constant (assuming a sufficient density of initial data).

However, Figure 3b shows that, for non-spherical objects, a single reference point results in increasingly large spacing between positions. This is because the vector between the origin and the surface rotates from near normal to highly oblique. At an oblique angle, a single data point will represent a large surface area, resulting in a low resolution model.

To maintain a nearly constant lateral resolution, more reference points should be used. Each reference point is used to validate the data that is closest to it. The user must select enough reference points to ensure that the vector between the object surface and the closest reference point does not become highly oblique.

**Figure 4:**
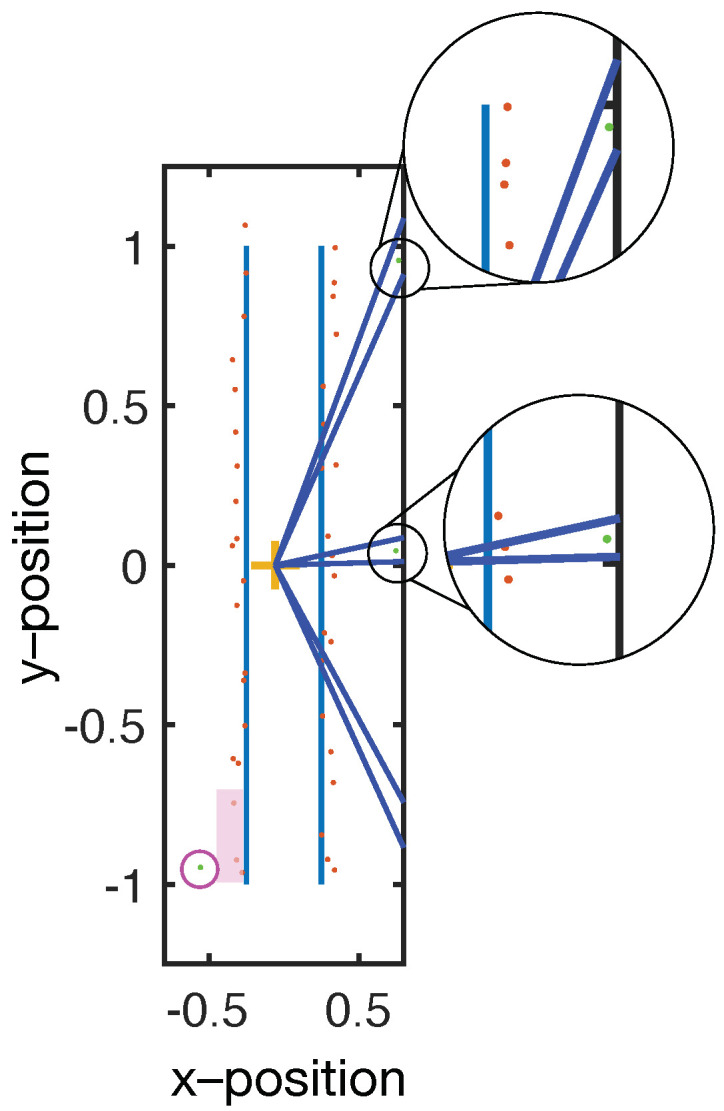
In irregular geometries or when the data density is low, outliers can be selected as valid, as shown in the top inset. The lower inset shows a case where an outlier is rejected. To prevent outliers from being selected, a coarser angular range is used to select points for which the mean distance to the reference is calculated (purple block). The mean is used to exclude points that are more than 20% greater than the mean (magenta circle).

A weakness of the filtering methodology described above is that if there is only a single data within an angular section, then it is automatically chosen. This can result in outliers that distort the final image (see Figure 4). To eliminate the outliers, a modified version of the same volume minimization algorithm is used. Instead of selecting only the closest points, the algorithm eliminates points that are more than 20% further away than the average of the data within that angular section. The outlier filter operates after the selection filter and at a coarser angular resolution than the selection filter so that sufficient data are used to calculate the average.

Note that, as shown in Figure 2b, the pipeline only passes validated data for visualization. However, the selection process always operates on the entire data set. In other words, if a user scans their hand over one section of an object and then later returns to that section, both the old and the newly acquired data points are included in the filtering process. Since the CAD software renders the point cloud in near real-time, the user is able to observe the build up of the model and can rescan areas where the data density is either too low or has many outliers.

## 3. Results

**Figure 5:**
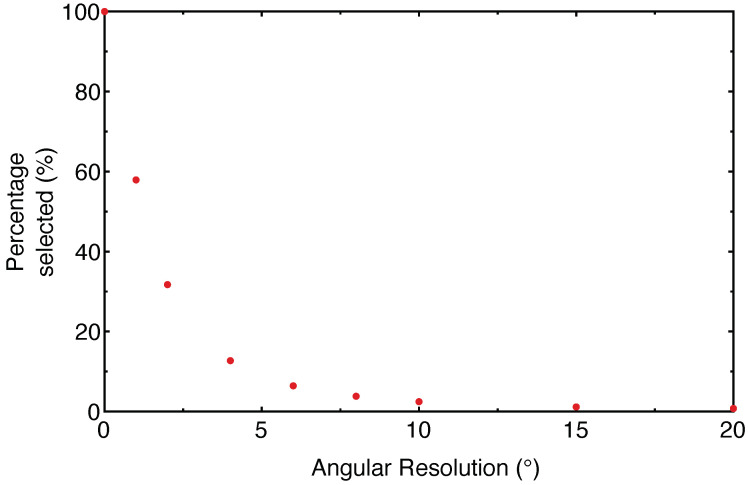
The selectivity of the filtering algorithm for increasing angular resolution. As can be seen, the percentage of retained data falls off sharply with coarser angular resolution.

The selectivity of the sensor was tested on a cylinder (approximately 7 cm in diameter and 20 cm high). As can be seen in Figure 5, the percentage of retained data drops sharply with coarser angular resolution.

It is important to note that the data selection process is a balance between three conflicting requirements. High spatial resolution images require that the angular resolution of the filter is maintained at a high value. However, if the angular resolution is too high, then the selectivity of the algorithm drops and the resulting virtual image is noisy. Furthermore, the time it takes for the filter to process the data increases, making it difficult to maintain real-time visualization. On the other hand, if the angular resolution is decreased, the filter operates faster and decreases the noise in the virtual image. However, the resolution of small features is compromised by the relatively poor angular resolution.

**Figure 6:**
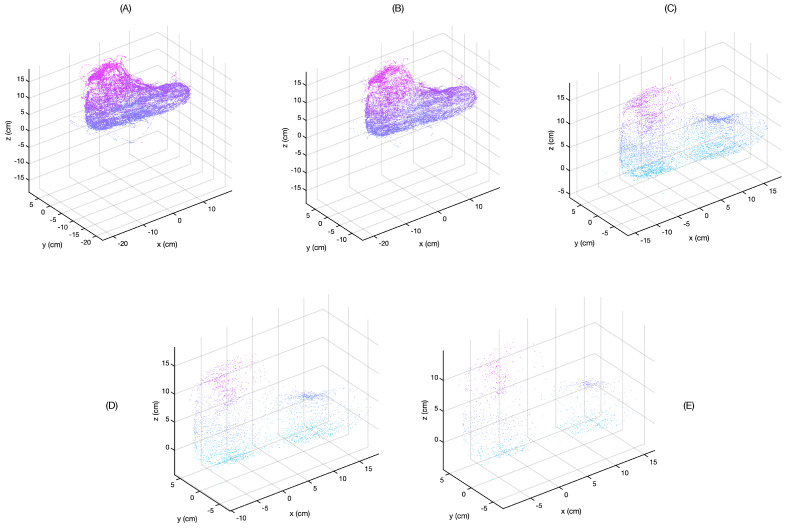
The effect of filtering. As the angular resolution of the filter decreases, the number and extremity of the outliers decreases. However, the total number of data points decreases sharply and the image resolution becomes poorer. The resolutions are, in order from (**A**–**E**): 0.25${}^{\circ }$, 1${}^{\circ }$, 3${}^{\circ }$, 5${}^{\circ }$, and 8${}^{\circ }$.

The quality of the resulting image was compared by scanning a model foot and applying different filter settings to the saved data. The result of this can be seen in Figure 6. For these data, two reference locations were set: one near the bridge of the foot and one near the ankle.

**Figure 7:**
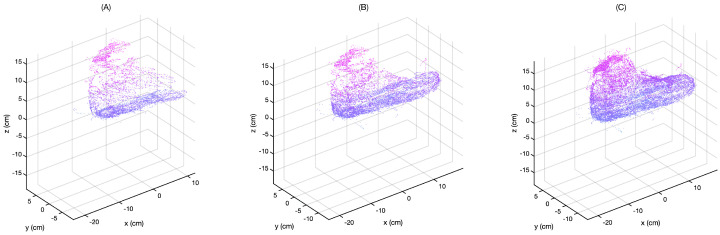
Build–up of visualization as the number of points increases from 0 to 10,000 (**A**), to 20,000 (**B**), and to 30,000 (**C**) at an angular resolution of 1${}^{\circ }$. The removal of points that do not lie on the surface is particularly notable on the bridge of the foot. In (**A**), the bridge consists of points that do not lie on the surface, while in (**B**,**C**) the bridge surface is better defined.

The improvement of the 3D model clarity over time is shown in Figure 7 for a resolution of 1${}^{\circ }$. Figure 7A–C shows the point cloud as the scan proceeds in time. The user is repeatedly moving their hand over the foot, adding new points for the selection algorithm to validate. As a result, outliers and points that are not on the surface are progressively removed.

**Figure 8:**
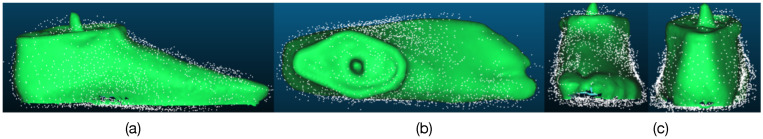
Comparison between optical imaging (green image) and scan data (point cloud). Sideview (**a**), top view (**b**), and front and back view (**c**). The two techniques agree within 3 mm in general with some outliers still selected as valid data.

A scan of a model foot (angular resolution of 4${}^{\circ }$, resulting in 2874 valid points) is compared with a standard optical imaging technique (occipital structure scanner, connected to an iPad) in Figure 8. It is clear that the resulting scan is reasonably close to that obtained by the optical imaging technique. It is notable that the data selection algorithm removes the vast majority of invalid data points, but it is not perfect. For example, the accumulation of data on the side of the foot in Figure 8c reduces the image accuracy.

**Figure 9:**
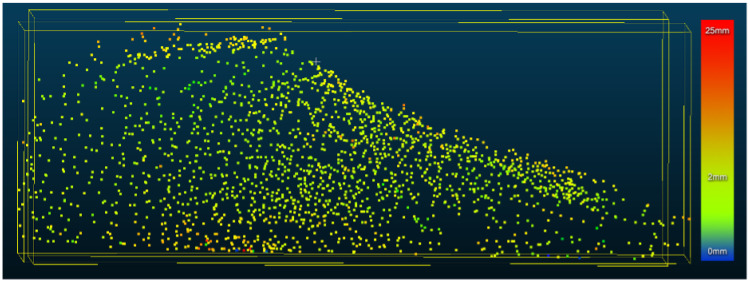
Point cloud of model foot taken with 4${}^{\circ }$ resolution. The color of the points indicates the distance between the point and the nearest mesh surface from an optical image of model foot (not shown for clarity). The average is 2.9 mm, but some outliers (∼25 mm) are still taken as valid data.

CloudCompare [12] was used to measure the distance between the mesh generated by the optical scan and the point cloud generated from the glove (see Figure 9). The average separation was found to be 2.9 mm. However, outliers, with a distance up to 25 mm, are also present.

## 4. Discussion

The smart glove presented here represents the beginning a new concept in the treatment of ankle-foot orthosis. The glove allows a 3D model of the foot to be built up while it is being manipulated by the clinician. The smart glove provides complete freedom, allowing the clinician to access the foot from all angles, as well as underneath the foot. By using a minimum volume algorithm to validate the data, the resulting model is within 3 mm of more commonly used optical imaging models. However, this is still large compared to the estimated accuracy of plaster casts (∼1 mm) that are currently used.

A portion of the error is due to the placement of the sensor and the lack of calibration. The sensor is placed on the glove and is not always in contact with the foot model, even when the fingers are in contact with the model. It is, therefore, important to add a calibration step to the software.

Nevertheless, even in its current form, the sensing glove is clinically useful when paired with an optical imaging technique. The smart glove data is validated by minimizing the distance to a reference point and thereby the volume of the shape. Many manipulations involve pressing the foot into a new shape *locally*, which reduces the distance to the reference point (and effectively reduces the volume). Thus, the resulting 3D model includes these manipulations by default. By comparing the optically obtained 3D model with the model obtained from the smart glove, the shape obtained from the clinician’s foot manipulation can be extracted from the difference between the two 3D images.

The goal of SmartScan is to replace the current process of creating a physical model with a new process in which a digital model is used. Under current practice, a plaster cast of the foot, which is held in the correct form while the plaster is setting, is used to obtain the surface shape of the correction. The surface shape must then be processed into a 3D model, which is finally produced and tested by the patient. The SmartScan system replaces the physical model with a digital model that allows the clinician to use the difference between the un-manipulated foot and the manipulated foot to determine the 3D shape of the required correction, which can then be directly produced and tested. Such a system has numerous clinical and educational advantages. First, test models can be printed locally for testing before a finished product is produced. By shortening the feedback cycle, a higher rate of success may be achieved. Second, the clinician has access to the model data, including their own movements, which they can use to adjust their own practice.

This second point may also have influence on training and education. Using SmartScan, students can compare their own manipulations of a foot with those of experienced professionals and then correct their process. With the present system, students can quickly see whether they are focusing on the right locations and are manipulating the foot in a similar manner to an experienced instructor. Once more sophisticated data analysis is available, the student will be able to compare their foot manipulation more accurately. For instance, with the addition of pressure sensors, students will be able to see whether the differences in final form are due to a lack of (or too much) pressure as opposed to focusing on the wrong part of the foot.

In order to achieve these goals, it must be possible to correctly classify the position data into three categories: data from when the glove is not in contact with the foot, data from when the glove is in contact with the foot and data from when the foot is held in the corrected position. In this paper, we have demonstrated that a volume minimization technique is effective in separating data into two categories: data from when the glove is not in contact with the foot and data from when the foot is in contact with the foot. However, the signal processing system does not yet distinguish between the natural shape of the foot and the corrected shape. Indeed, the current filtering technique will provide the points that are closest to the reference locations, meaning that over a complete dataset, it is likely to only provide the corrected shape and not the natural shape of the foot.

To further distinguish between the natural and corrected shape of the foot, several approaches may be used. Our current work focuses on using additional sensors (i.e., pressure sensors), which may be used to sort the position data, and machine learning algorithms, which may be used to sort the data based on a training set of classified data.

## 5. Conclusions

In conclusion, we have presented a smart glove designed to help orthotists model the shape of implants required to correct ankle-foot orthosis. To do this, the smart glove incorporates absolute position sensors to provide absolute position and orientation of the hand as a function of time. By assuming that the glove is manipulating a minimum volume object (the foot), the shape of the foot can be obtained by sequentially removing the points furthest away from the center of mass of the points (a reference location). When the orthosist bends the foot into the right shape, the final orientation is naturally included in the data because these locations are closer to the center of mass than the natural orientation of the foot.

## Figures and Tables

**Table 1:** Software blocks for sensor data collection, filtering and visualization.

Block	Description
RhinoCAD	CAD software
SmartScan Rhino plug-in	RhinoCAD plug-in for measurement control and visualization
Rhino API	Software library used to interface with the RhinoCAD application
SmartScan CLI	Command line interface for the SmartScan system
SmartScanService	The SmartScan software library, implemented as a C++ class
TrakSTAR controller	Class that interfaces with the TrakSTAR driver
Scan	Class encapsulating the measurement and filtering pipeline
Filtering	Class implementing the filtering algorithms
ReferencePoint	Custom datatype for reference points
CSV Export	Class for formatting and exporting the data
TrakSTAR API	Software library facilitating TrakSTAR driver communication
TrakSTAR Driver	Microsoft Windows driver for the TrakSTAR device

## Data Availability

Code and data are available at https://github.com/exMamaku/smart-scan (accessed on 8 April 2021).
